# Inorganic polyphosphate in mammals: where's Wally?

**DOI:** 10.1042/BST20190328

**Published:** 2020-02-12

**Authors:** Yann Desfougères, Adolfo Saiardi, Cristina Azevedo

**Affiliations:** Medical Research Council Laboratory for Molecular Cell Biology, University College London, London, U.K.

**Keywords:** cell metabolism, inorganic polyphosphates, phosphate, post translational modification, signalling

## Abstract

Inorganic polyphosphate (polyP) is a ubiquitous polymer of tens to hundreds of orthophosphate residues linked by high-energy phosphoanhydride bonds. In prokaryotes and lower eukaryotes, both the presence of polyP and of the biosynthetic pathway that leads to its synthesis are well-documented. However, in mammals, polyP is more elusive. Firstly, the mammalian enzyme responsible for the synthesis of this linear biopolymer is unknown. Secondly, the low sensitivity and specificity of available polyP detection methods make it difficult to confidently ascertain polyP presence in mammalian cells, since in higher eukaryotes, polyP exists in lower amounts than in yeast or bacteria. Despite this, polyP has been given a remarkably large number of functions in mammals. In this review, we discuss some of the proposed functions of polyP in mammals, the limitations of the current detection methods and the urgent need to understand how this polymer is synthesized.

## Introduction

Arthur Kornberg and Igor Kulaev spent a large part of their careers working on inorganic polyphosphate (polyP). Convinced of the fundamental importance of this ‘forgotten polymer', they and their teams developed tools to study polyP in a wide range of organisms from bacteria to mammals [1,2]. Today, polyP is recognized as a molecule of many and important functions. The large spectrum of activities in medically relevant contexts have made polyP a highly promising target for therapeutic applications. In recent years, a large number of publications have reported on the diverse effects of exogenously added synthetic polyP to cultured cells. Unfortunately, the complete lack of information on the biosynthetic machinery of mammalian polyP has hampered our understanding of the physiological relevance of this polymer in mammals. The discovery of the human polyP synthase would validate these previous findings and dramatically accelerate polyP research. However, this may be a herculean task considering that to date, more than 70 years after Arthur Kornberg and Igor Kulaev, started working on polyP, no enzyme carrying the ability to synthesize polyP in mammals has been identified.

## The enzymes that metabolize polyP

Despite the presence of polyP in all tested organisms from bacteria to mammals, the enzymology responsible for its synthesis is not conserved. Bacteria have two highly conserved enzymes capable of synthesising polyP, PPK1 and PPK2. PPK1 forms a tetramer, of 80 kDa units with structural similarities to Phospholipase D [3] but without defined domains, that catalyses the reversible transfer of ATP's gamma-phosphate to polyP [4]. PPK2 is a dual activity 40.8 kDa protein that synthesizes polyP from GTP or ATP [5] and can use polyP as a donor to convert GDP to GTP [6]. In *Dictyostelium discoideum*, two PPK activities have been originally reported, one with sequence similarity to the bacterial PPK1 that was acquired through horizontal gene transfer [7], and a novel DdPPK2 activity, that seems to be a complex of three actin-related proteins: Arp1, Arp2, and an unreported Arpx [8]. No genetic data is available confirming DdPPK2 polyP kinase activity. However, in contrast with what was previously demonstrated [9], we recently reported that *D. discoideum* cells lacking DdPpk1 have no detectable polyP levels demonstrating that this is the sole enzyme responsible for polyP synthesis in social amoeba [10]. In fungi and several other protists, including *Trypanosoma* and *Leishmania*, polyP synthesis is performed by a different enzymatic complex associated with the membrane-integral vacuolar transporter chaperone (VTC) complex [11,12]. Here polyP is synthesized from ATP on the cytosolic face of acidocalcisome-like organelles and translocated into the lumen, a process requiring the proton gradient generated by the V-ATPase [13].

Another set of enzymes is responsible for the hydrolysis of polyP. These proteins can be classified as either endopolyphosphatases, if they degrade polyP by attacking internal phosphoanhydride bonds, or exopolyphosphatases, if they hydrolyse the terminal phosphate residue of the polymer. In bacteria, the exopolyphosphatase Ppx1 is the only characterized polyP hydrolase. This enzyme is conserved in fungi where it prevents the accumulation of cytosolic polyP [13] which, in excess is toxic. Ppx1 is a member of a broader family of phosphoesterases called DHH [14], which includes the mammalian homologue H-Prune [15]. This enzyme shares many similarities with bacterial Ppx1 and *in vitro* assays confirmed that it has strictly exopolyphosphatase activity [16]. Another potent exopolyphosphatase is alkaline phosphatase from calf intestine [17]. Yeast and mammals also possess endopolyphosphatases [18–20]. In *S. cerevisiae*, this arsenal is composed of two vacuolar-localized proteins named Ppn1 and Ppn2, and an additional enzyme localized in the cytosol and the nucleus called Ddp1. Ddp1 is a homologue of human DIPP proteins, members of the Nudix family, that also exhibit endopolyphosphatase activity [18]. Therefore, although no polyP synthase has been identified in mammals, two enzymes able to degrade polyP have been characterized, an exo- and an endopolyphosphatase. Interestingly, there are also proteins, like the NAD kinase, able to use polyP instead of ATP as phosphate donor [21].

## Functions of polyP in mammals

**Figure 1.**
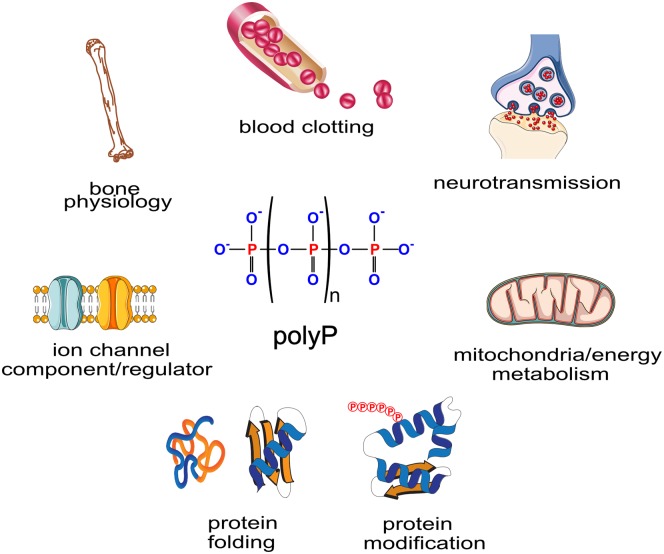
polyP structure and its attributed functions in mammals. The polyP chemical formula is represented centrally, where ‘n’ can range from 1 to several hundreds. Thus a phosphate chain with at least three phosphate groups can be considered polyP. Surrounding the chemical formula are graphical representations of the different functions attributed to polyP in mammals.

Despite the lack of knowledge of the biosynthetic pathway for polyP synthesis and hence of a direct way to genetically manipulate its levels in mammalian cells, it is remarkable how much is known about its functions, and how diverse these are (Figure 1). Initially detected in platelets [22] its role in blood coagulation and in the inflammatory response was soon established [23]. PolyP regulates several points in the blood-clotting cascade [24,25]. The different actions of polyP in this context seem to depend on the length of the polymer [25], adding unexpected complexity beyond the simplicity of its molecular structure. Therefore, polyP is a strong candidate to develop new therapeutic approaches to modulate blood clotting. Another area of intense research focuses on the role of polyP in bone and cartilage formation. Here again, polyP has been shown to act at different places to strategically promote the regeneration of these tissues [26,27].

Several studies have directly or indirectly demonstrated that polyP is somehow involved in cancer progression. Human MCF-7 breast cancer cells overexpressing the yeast exopolyphosphatase Ppx1 have reduced growth in serum-free media and are deficient in their response to mitogens, as they fail to activate mTOR [28]. The human exopolyphosphatase H-Prune, is often up-regulated in metastatic cancers, and is associated with cancer progression, tumour aggressiveness and advanced disease status in breast and gastric cancer [16]. PolyP has been found in substantially higher levels in myeloma cells compared with normal primary plasma cells, B cells and non-myeloma bone marrow mononuclear cells [29]. Lastly, exogenously-added polyP induces apoptosis specifically in myeloma cells and plasma cells [30].

PolyP has also been identified as a component or a regulator of membrane channels. It associates with the non-selective mitochondria permeability transition pore (mPTP) complex in the inner mitochondrial membrane [31], and with the transient receptor potential channels melastatin (transient receptor potential channel subfamily M, TRPM8) [32] or with TRPA1 (transient receptor potential channel, subfamily A, member 1) [33]. Its association with calcium (Ca^2+^) and the polymer polyhydroxybutyrate (PHB), seems to be sufficient to produce a protein-free channel after insertion into lipid bilayers. This structure would be able to selectively transport calcium. PolyP and PHB have both been found to interact with TRPM8, modulating the activity of this cation channel [32]. The exact role of these associations is not entirely understood: polyP might contribute to ion-conduction through an electrostatic interaction with these proteins, helping to maintain an active conformation for channel gating, or through covalent modification of the protein by polyP (see below). Due to its affinity for calcium, polyP is also believed to buffer calcium availability within mitochondria. Free phosphate is known to play an essential role in binding calcium in the mitochondrial matrix to reduce the free calcium concentration. By artificially tagging the expression of Ppx1 to the mitochondrial matrix, the laboratory of E. Pavlov showed that polyP increases free calcium concentration, supposedly by decreasing the amount of calcium-phosphate precipitates [15]. A further link to mitochondrial function was recently demonstrated showing that polyP synthesis was dependent on the metabolic status of the cell, and that polyP levels correlate with cellular ATP levels [34]. Altogether, these data attribute a central role for polyP in regulating energy metabolism.

PolyP has also been described as a gliotransmitter. Upon calcium stimulation polyP is released from astrocytes (and a small proportion of neurons) and can subsequently be taken up by neurons [35]. While in the intercellular medium, polyP activates P2Y receptors, which stimulate PLC-dependent synthesis of IP_3_. This ultimately results in the release of calcium from the endoplasmic reticulum. However, the exact role of polyP in neuronal signalling remains unknown.

Recently, further functions have been attributed to polyP. First, the discovery that polyP is a molecular chaperone in bacteria, stimulated the search for a function in pathological protein aggregation [36]. This work, undertaken by Ursula Jakob's laboratory, demonstrated that polyP nucleates fibril formation in Abeta peptide, Tau, alpha-synuclein, and huntingtin proteins involved in protein-folding diseases such as Alzheimer's, Parkinson's and Huntington's [37,38]. This nucleation is thought to decrease the abundance of the soluble toxic intermediate species. Second, our laboratory determined the ability of polyP to covalently modify proteins in a process called polyphosphorylation [39]. This new post-translational modification occurs by attachment of the polymer to a lysine residue, conferring to polyP a novel role in signal transduction. Two genome-wide screens last year identified a number of human proteins susceptible to be polyphosphorylated [40,41].

## Detection and quantification of polyP in mammalian cells and tissues

The high abundance of polyP in micro-organisms facilitates its detection and quantification. Mammalian cells possess much lower amounts of the polymer and more sensitive methods, such as [^32^P] radiolabelling, are therefore required. In mammals, polyP was first reported in 1963 and its characterization was performed using a set of biochemical assays. Nowadays, besides these classical assays, the semi-quantitative detection of polyP is achieved using microscopy after staining with fluorescently tagged proteins or fluorescent dyes. These approaches are now reviewed and discussed.

### Early biochemical analysis

Early studies on polyP used subcellular fractionation and biochemical assays for precise quantification. After isolation of the organ/organelle, the polyP extraction was usually performed by precipitating the proteins and releasing polyP through boiling on concentrated bases. PolyP would then be further purified by precipitation or separation on affinity columns. The resulting material would be analysed by thin-layer chromatography for its metachromatic potential, or by nuclear magnetic resonance. Quantification was often performed by orthophosphate measurement after hydrolysis in strong acid. Using these methodologies, the presence of polyP in mammalian cells was first reported in 1963 by Lynn and Brown who demonstrated that its synthesis was stimulated in rat liver mitochondria by succinate, a mitochondria swelling agent [42]. In the same year, polyP, of an estimated chain length of 2 to 500 units, was also shown to be present in rat liver nuclei [43]. In rat, the liver was estimated to have 1–2 µg of polyP per gram of tissue [44] whereas an adult brain contains a much higher amount, 15 µg [43]. In bovine, polyP was present in all tissues tested (brain, liver, pancreas, kidney cortex and spleen) with an estimated chain length between 10 to 5000 units [43]. The same study also reported the presence of polyP in rabbit erythrocytes, with the major proportion co-fractionating with membranes, suggesting that the presence of a cell nucleus is not an absolute requirement for the maintenance of a high polyP content. Although the procedures used in these studies paved the way for analysing polyP in mammalian cells, they rely on harsh extraction conditions and a non-specific quantitative assay based on the lability of the phosphoanhydride bond. These crude methods have likely led to a misattribution of the final readout, in the form of free phosphate amount, exclusively to polyP. Although the use of [^32^P] allows detection of lower polyP amounts, it also requires the use of defined media with low phosphate, a condition that, as demonstrated in micro-organisms, favours degradation of polyP rather than its synthesis. To overcome these drawbacks, other approaches were later developed and optimized: extraction procedures involving acidic phenol or guanidine isothiocyanate, and specific enzymatic assays reducing the need for the polyP extract to be of the highest purity. The bacterial exopolyphosphatase Ppx1 is now routinely used to degrade polyP *in vitro*. The orthophosphate residues released are then quantified by standard assays such as the malachite green. Alternatively, the reverse reaction of the polyphosphate kinase Ppk1, consisting of the transfer of phosphate from polyP to ADP, generates ATP that can be quantified with the sensitive luciferin/luciferase assay. Finally, the use of polyacrylamide gel electrophoresis (PAGE) is critical in the definitive identification of polyP, as it allows the detecting of a ladder like appearance, consequent of the stepwise increases of polyP's polymeric nature. PAGE is also essential for the estimation of the chain length of the polymer. The omission of urea from the PAGE recipe [45] considerably increases the resolution by allowing the use of gels with higher polyacrylamide concentration. PolyP detection within the gel is achieved by staining with the high molecular mass dye Toluidine Blue or the fluorescent dye DAPI. In the past two decades, with these new polyP purification methods and detection technologies, several labs have investigated the subcellular presence of polyP, and its tissue and cell line distribution. PolyP has been detected from mouse fibroblast (NIH 2T3), monkey kidney (Vero), human lymphoma (Jurkat), human embryonal kidney (193/E1A), mouse mammary gland (Mouse L) and rat adrenal phechromocytoma (PC12) cells, rat liver nucleus, plasma membrane, cytosol, mitochondria, microsomes [46], lysosomes of human fibroblasts [47], nucleolus of human myeloma cells [29], and human platelets [22]. For unknown reasons, it seems that the neural polyP is extremely labile to catabolic degradation after death whereas the polyP found in the liver is very stable [43].

### The rise of microscopy

Using microscopy to visualize polyP has several advantages. For one, it does not require the convoluted extraction procedures explained above. It also allows determination of the subcellular localization of the polymer, a difficult task when using classical sub-cellular fractionation approaches and [^32^P] labelling. The most widespread dye used to visualize polyP is DAPI, which is better known for its nucleic acid binding properties. However, the ability of DAPI to bind to a large variety of molecules may hamper the detection of low amounts of polyP as in mammalian cells. Recently, more specific fluorescent dyes found to bind polyP (JC-D7 and JC-D8) allowed its visualization in living mammalian cells [48]. However, these compounds also bind to heparin, another negatively charged polymer, with almost equal affinity as polyP. Alternatively, the use of the polyP binding domain of Ppx1 (PPBD) coupled to the Green Fluorescent Protein (GFP) has also been reported [49,50]. Although this procedure requires fixing of the cells prior to incubation with the reporter, it is believed to be more specific towards polyP. Microscopy does not allow for an absolute quantification of the polyP due to heterogeneous nature of this polymer and all the dyes have different affinities for polyP according to the chain length. For most of these procedures using microscopy there is the need to fix the cells which requires extensive washing steps that may remove a substantial amount of polyP from the cells. Although microscopy has become the most popular, and unfortunately sometimes the only, technique used to detect polyP in mammalian cells, the genetic proof to ascertain polyP presence is lacking.

## Beyond observation: the search for a polyP synthase activity

Although initial studies aimed at detecting an enzymatic activity able to synthesize polyP, researchers are now focused on describing the phenotypes induced by simply adding synthetic polyP to the culture medium. The only hint reported of a possible polyP synthase in mammalian cells came from Reusch et al. twenty years ago [51]. The authors suggested that the plasma membrane calcium pump ATP2B1, purified from human erythrocytes, may function as polyP kinase by exhibiting ATP-polyphosphate transferase and polyphosphate-ADP transferase activities. Unfortunately, this old observation lacks further confirmation.

## Perspectives

- PolyP is a ubiquitous molecule with very important and diverse roles in biology, regulating a remarkable breadth of physiological functions that encompass research from haematology to neuroscience.
- The current scientific enterprise is focussed on fast and flashy publications that somehow seems to have forgotten that the mechanism of polyP synthesis in mammals is yet to be identified and characterized. While it is easy to observe the effect of adding polyP to *in vitro* assays or to culture media, these are mere phenomenological observations.
- The discovery of the polyP synthase in mammalian cells will be of instrumental importance to validate all these observations. This will require classical biochemical fractionations and assays likely requiring the use of radiolabelled [^32^P]-ATP to improve sensitivity. Therefore, the quest to find the mammalian polyP synthase is still open and for the health of the research field must be accomplished soon. Only then might we be able to confidently say not only where Wally is, but also what are its actual physiological functions.

## Abbreviations

ATP: adenosine triphosphate
GTP: guanosine triphosphate
polyP: inorganic polyphosphate
PPBD: polyphosphate binding domain
Ppk: polyphosphate kinase
Ppn: endopolyphosphatase
Ppx: exopolyphosphatase
VTC: vacuolar transporter chaperone complex

## Open Access

Open access for this article was enabled by the participation of University College London in an all-inclusive *Read & Publish* pilot with Portland Press and the Biochemical Society under a transformative agreement with JISC.
